# Whole-Body Imaging of Neural and Muscle Activity during Behavior in *Hydra vulgaris*: Effect of Osmolarity on Contraction Bursts

**DOI:** 10.1523/ENEURO.0539-19.2020

**Published:** 2020-08-20

**Authors:** Wataru Yamamoto, Rafael Yuste

**Affiliations:** 1 Neurotechnology Center, Department Biological Sciences, Columbia University, New York, NY 10027; 2 Marine Biological Laboratory, Woods Hole, MA 02543

## Abstract

The neural code relates the activity of the nervous system to the activity of the muscles to the generation of behavior. To decipher it, it would be ideal to comprehensively measure the activity of the entire nervous system and musculature in a behaving animal. As a step in this direction, we used the cnidarian *Hydra vulgaris* to explore how physiological and environmental conditions alter simple contractile behavior and its accompanying neural and muscle activity. We used whole-body calcium imaging of neurons and muscle cells and studied the effect of temperature, media osmolarity, nutritional state, and body size on contractile behavior. In mounted *Hydra* preparations, changes in temperature, nutrition state, or body size did not have a major effect on neural or muscle activity, or on contractile behavior. But changes in media osmolarity systematically altered contractile behavior and foot detachments, increasing their frequency in hypo-osmolar media solutions and decreasing it in hyperosmolar media. Similar effects were seen in ectodermal, but not in endodermal muscle. Osmolarity also bidirectionally changed the activity of contraction burst (CB) neurons, but did not affect the network of rhythmic potential (RP) neurons in the ectoderm. These findings show osmolarity-dependent changes in the activity of CB neurons and ectodermal muscle, consistent with the hypothesis that CB neurons respond to media hypo-osmolarity, activating ectodermal muscle to generate CBs. This dedicated reflex could serve as an excretory system to prevent osmotic injury. This work demonstrates the feasibility of studying an entire neuronal and muscle activity in a behaving animal.

## Visual Abstract

**Figure:**
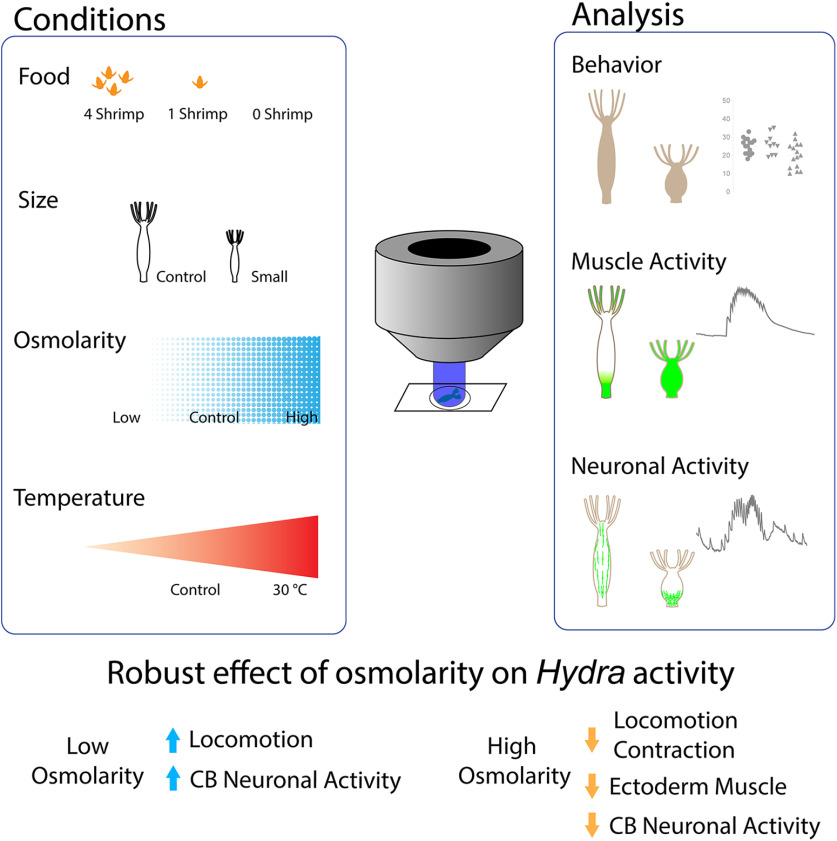

## Significance Statement

We imaged whole-body muscle and neuronal activity in *Hydra* in response to different physiological and environmental conditions. Osmolarity bidirectionally altered *Hydra* contractile behavior in a reflexive fashion. These changes were accompanied by specific changes in the activity of one neuronal circuit and one set of muscles. By providing neurobiological mechanisms for a reflex in a cnidarian, this work is a step toward comprehensive deciphering of the mechanisms of animal behavior by measuring the activity of all neurons and muscle cells.

## Introduction

Calcium imaging of neuronal circuits (Yuste and Katz, 1991) has enabled recent investigations of the circuit basis of animal behavior in a number of transparent organisms such as *Caenorhabditis elegans*, *Drosophila* larvae, and zebrafish embryos (Nagel et al., 2005; Liewald et al., 2008; Honjo et al., 2012; Cong et al., 2017; Kim et al., 2017). While these studies have focused on particular parts of the nervous system, to systematically understand the neural code, i.e., the relation between the activity of a nervous system and behavior, it would be ideal to measure the activity of the entire nervous system and the entire muscular tissue during the entire behavioral repertoire of an animal. This is now possible with the transparent fresh-water cnidarian *Hydra vulgaris*, using transgenic strains that express calcium indicators in every neuron (Dupre and Yuste, 2017) and every muscle cell of the body (Szymanski and Yuste, 2019), and applying machine learning to systematically analyze its behavior (Han et al., 2018). *Hydra* has a simple body consisting of ectoderm and endoderm myoepithelial cells. Muscular processes, myonemes, run longitudinally in the ectoderm and radially in the endoderm. Thus, each myoepithelial layer can have distinct functions in different behaviors, but can also become coactive during sustained contractions (Szymanski and Yuste, 2019).

*Hydra* has one of the simplest nervous system in evolution, with several hundreds to a few thousand neurons, depending on the size of the animal (Hadzi, 1909; Parker, 1919; Westfall et al., 1991). The simplicity of *Hydra*’s system gives hope that systematic measurements of the neural and muscular activity of behaving *Hydra* could be used to decipher the mechanisms of behavior. *Hydra* neurons are believed to be multifunctional. A sensory neuron with sensory cilia also synapses with epithelial cells as a motor neuron (Westfall, 1973). These neurons are organized in two independent nerve nets, in the ectoderm and endoderm (Dupre and Yuste, 2017). *Hydra*’s nerve nets are distributed throughout the body of the animal, without any cephalization (Epp and Tardent, 1978). Several independent neuronal circuits, interspersed within the nerve nets, are active synchronously in an oscillating manner. The main ones named contraction burst (CB) and rhythmic potential (RP)1 circuits, involve independent groups of ectoderm neurons, whereas a third circuit, the RP2 circuit, involves endodermal cells (Dupre and Yuste, 2017). These three circuits are associated with three different motor behaviors: CBs (CB circuit), elongation (RP1), and egestion (RP2; Dupre and Yuste, 2017).

*Hydra* is a fresh-water animal living in ponds, lakes and streams. Because of this, *Hydra* experiences fluctuations in temperature and osmolarity as well as the amount of food available, which determines its body size. Previous research has described *Hydra* responses to changes in environmental and physiological conditions. Those include decreases in contractions with increased osmolarity (Benos and Prusch, 1973) and after feeding (Grosvenor et al., 1996; Rushforth and Hofman, 1972) and necrosis after acute increases in temperature (Bosch et al., 1988). These past studies show that external modification of *Hydra* behavior is possible.

Motivated by this work, we explored systematically how different environmental conditions affect *Hydra* behavior, focusing on body contractions. Do do so, we performed measurements of *Hydra* behavior under standard conditions in mounted and freely behaving animals and used calcium imaging to measure how neurons and muscular cells responds to physiological and environmental conditions important for their survival. Experimental conditions included high or low osmolarity (control, 50 mm sucrose or diH_2_O), temperature (23°C or 30°C), food (zero, one, and four shrimp per day for a week), and body size (mature vs newly released buds). In each of these conditions, we measured the number of contractions and foot detachments in behavior assays, the ectodermal and endodermal muscle activity, and the activity of the CB and RP1 neuronal circuits.

We expected to see major changes in behavior, neuronal, and muscle activity, as the chosen conditions are essential to *Hydra* survival. But surprisingly, in mounted preparations, we only found robust effects due to osmolarity. Increased osmolarity decreased contractions frequency, consistent with Benos and Prusch (1973), decreased foot detachments and also decreased the activity of CB neurons and ectodermal muscle cells, whereas decreased osmolarity had opposite effects, as a reflex. Our results indicate that *Hydra*’s CB circuit senses osmolarity to control ectodermal muscle and generate contractile behaviors, revealing a specific neuro-muscular reflex that probably evolved for osmoprotection.

## Materials and Methods

### Materials

Sucrose and sea salt were purchased from Sigma. Brine shrimp, *Artemia nauplii*, were obtained from Brine Shrimp Direct. We used transgenic *Hydra* expressing GCaMP6s in neurons (Dupre and Yuste, 2017) or in ectoderm/endoderm muscle cells (Szymanski and Yuste, 2019).

### *Hydra* culture

*Hydra* were maintained in media composed of 1.3 mm CaCl_2_, 0.02 mm MgCl_2_, 0.03 mm KNO_3_, 0.5 mm NaHCO_3_, and 0.08 mm MgSO_4_ in an 18°C incubator. *Hydra* were fed brine shrimp three times a week and were starved for 2 d before an experiment.

### Environmental or physiological conditions

The following conditions were used. (1) Food: *Hydra* were fed zero, one, or four shrimp every day for a week. *Hydra* were starved for 1 d before an experiment. (2) Size: *Hydra* with large (∼1 cm) or small (∼0.3 mm) sizes, chosen after bud separation, were fed once. (3) Temperature: room (23°C) or high temperature (30°C). (4) Osmolarity: *Hydra* were imaged in media with low osmolarity (diH_2_O, 0 mOsm/l), control medium (control, *Hydra* media, 5 mOsm/l, fresh water is usually between 2 and 8 mOsm/l), or high (50 mm sucrose, 50 mOsm/l) osmolarity.

### Calcium imaging

Wide-field calcium imaging of *Hydra* was conducted at 2 Hz using a fluorescence dissecting microscope (Leica M165) equipped with a long-pass GFP filter set (Leica filter set ET GFP M205FA/M165FC), 1.63× Plan Apo objective, and a sCMOS camera (Hamamatsu ORCA-Flash 4.0). A mercury arc lamp was used to illuminate the sample. *Hydra* were mounted between coverslips with 100- to 200-μm spacers, depending on animal thickness. All imaging was conducted at a room temperature ∼23°C unless indicated.

### Behavior analysis

The number of contractions and foot detachments were manually scored from calcium imaging movies (mounted *Hydra* between coverslips) or movies of freely moving *Hydra* in glass-bottom dishes (MatTek). Five animals were placed per well (depth is 700–750 μm) for 1-h recordings.

### Analysis of neural and muscular activity

Values for whole-body fluorescent intensity in each frame over time were obtained with ImageJ and used to detect CB and RP1 pulses using a semi-automated program in MATLAB. Whole-body muscle activity was analyzed in the same manner.

### Analysis of body column width

*Hydra* were imaged at 0.5 Hz using a dissecting microscope (Leica M165), 1.63× Plan Apo objective, and sCMOS camera (Hamamatsu ORCA-Flash 4.0). *Hydra* were mounted between coverslips with around 200-μm spacer in control media or in high-osmolarity solution (50 mm sucrose). To measure width, the body column of *Hydra* was fitted into ellipse using a program written by MATLAB. The lowest values from each cycle were used to calculate average width at the end of the elongation.

### Statistical methods

Data are shown as average ± SEM in figures and in the text. Two-tailed unpaired Student’s *t* test or one-way ANOVA with Tukey’s multiple comparison test were conducted in GraphPad Prism software (Table 1).

**Table 1:** Statistical tests and results

Figure	Description	Methods	95% CI of difference	Significant	*p* value
1*B*	Food: 0 vs 1	1	–2.355 to 6.718	No	0.4707
	Food: 0 vs 4	1	–3.537 to 5.537	No	0.8506
	Food: 1 vs 4	1	–5.718 to 3.355	No	0.7981
	Osmo: Ctr vs low	1	–6.364 to 4.864	No	0.9432
	Osmo: Ctr vs high	1	0.2450 to 10.25	Yes	0.038
	Osmo: Low vs high	1	0.3148 to 11.69	Yes	0.0367
	Size: Ctr vs small	2	–9.991 to –2.937	No	0.0008
	Temp: Ctr vs high	2	–0.5233 to 6.023	No	0.0958
1*C*	Food: 0 vs 1	1	–2.198 to 1.335	No	0.8207
	Food: 0 vs 4	1	–1.448 to 2.085	No	0.898
	Food: 1 vs 4	1	–0.9775 to 2.478	No	0.5411
	Osmo: Ctr vs low	1	–2.688 to 0.3822	No	0.1728
	Osmo: Ctr vs high	1	1.034 to 3.682	Yes	0.0003
	Osmo: Low vs high	1	1.958 to 5.064	Yes	<0.0001
	Size: Ctr vs small	2	0.08979 to 2.894	Yes	0.0378
	Temp: Ctr vs high	2	–0.9724 to 1.722	No	0.5716
1*E*	Food: 0 vs 1	1	–1.740 to 2.407	No	0.9195
	Food: 0 vs 4	1	0.3931 to 4.540	Yes	0.0164
	Food: 1 vs 4	1	0.05976 to 4.207	Yes	0.0426
	Osmo: Ctr vs low	1	0.7542 to 6.579	No	0.01
	Osmo: Ctr vs high	1	–0.2806 to 4.642	No	0.0925
	Osmo: Low vs high	1	–3.947 to 0.9758	Yes	0.3223
	Size: Ctr vs small	2	4.300 to 21.17	No	0.0059
	Temp: Ctr vs high	2	–6.122 to –2.412	Yes	<0.0001
1*F*	Food: 0 vs 1	1	–1.026 to 0.09217	No	0.1178
	Food: 0 vs 4	1	–1.426 to –0.3078	Yes	0.0014
	Food: 1 vs 4	1	–0.9588 to 0.1588	No	0.2029
	Osmo: Ctr vs low	1	1.610 to 3.724	Yes	<0.0001
	Osmo: Ctr vs high	1	0.6877 to 2.474	Yes	0.0002
	Osmo: Low vs high	1	–1.979 to –0.1925	Yes	0.0134
	Size: Ctr vs small	2	–0.1413 to 0.9413	No	0.1413
	Temp: Ctr vs high	2	–2.683 to –0.9838	Yes	<0.0001
2*C*	Food: 0 vs 1	1	–176.2 to 327.3	No	0.8033
	Food: 0 vs 4	1	–257.1 to 281.1	No	0.9991
	Food: 1 vs 4	1	–315.3 to 188.2	No	0.8705
	Osmo: Ctr vs low	1	–147.0 to 148.8	No	0.9998
	Osmo: Ctr vs high	1	12.02 to 307.8	Yes	0.0356
	Osmo: Low vs high	1	–6.375 to 324.4	No	0.0588
	Size: Ctr vs small	2	–138.6 to 167.4	No	0.8303
	Temp: Ctr vs high	2	–132.3 to 152.1	No	0.8738
2*D*	Food: 0 vs 1	1	–318.4 to 280.4	No	0.981
	Food: 0 vs 4	1	–473.7 to 125.1	No	0.2655
	Food: 1 vs 4	1	–475.4 to 164.8	No	0.378
	Osmo: Ctr vs low	1	–174.6 to 237.8	No	0.9107
	Osmo: Ctr vs high	1	–106.8 to 282.0	No	0.4681
	Osmo: Low vs high	1	–150.2 to 262.2	No	0.7494
	Size: Ctr vs small	2	2.523 to 332.7	Yes	0.0473
	Temp: Ctr vs high	2	–20.35 to 199.1	No	0.0955
2*E*	Food: 0 vs 1	1	–13.68 to 17.47	No	0.939
	Food: 0 vs 4	1	–19.72 to 15.75	No	0.948
	Food: 1 vs 4	1	–20.83 to 13.08	No	0.8034
	Osmo: Ctr vs low	1	–19.22 to 0.7527	No	0.0686
	Osmo: Ctr vs high	1	–6.431 to 13.54	No	0.5872
	Osmo: Low vs high	1	1.625 to 23.96	Yes	0.0273
	Size: Ctr vs small	2	–7.207 to 13.24	No	0.5081
	Temp: Ctr vs high	2	–4.729 to 13.86	No	0.2836
2*F*	Food: 0 vs 1	1	–19.97 to 13.83	No	0.9455
	Food: 0 vs 4	1	–30.51 to 3.289	No	0.1296
	Food: 1 vs 4	1	–28.61 to 7.526	No	0.3429
	Osmo: Ctr vs low	1	–18.81 to 7.069	No	0.4634
	Osmo: Ctr vs high	1	–7.909 to 16.49	No	0.6216
	Osmo: Low vs high	1	–2.777 to 23.11	No	0.1307
	Size: Ctr vs small	2	2.745 to 22.78	Yes	0.0188
	Temp: Ctr vs high	2	0.5891 to 18.07	Yes	0.0396
2*G*	Food: 0 vs 1	1	–1.613 to 1.854	No	0.9773
	Food: 0 vs 4	1	–0.8072 to 2.899	No	0.2839
	Food: 1 vs 4	1	–0.8077 to 2.659	No	0.3176
	Osmo: Ctr vs low	1	0.5862 to 3.721	Yes	0.0108
	Osmo: Ctr vs high	1	1.379 to 4.514	Yes	0.0017
	Osmo: Low vs high	1	–0.9592 to 2.545	No	0.4373
	Size: Ctr vs small	2	–7.207 to 13.24	No	0.5081
	Temp: Ctr vs high	2	–4.729 to 13.86	Yes	0.2836
2*H*	Food: 0 vs 1	1	–2.115 to 3.010	No	0.8669
	Food: 0 vs 4	1	–2.517 to 2.607	No	0.9985
	Food: 1 vs 4	1	–3.142 to 2.336	No	0.9032
	Osmo: Ctr vs low	1	–0.4909 to 5.189	No	0.1103
	Osmo: Ctr vs high	1	–2.518 to 2.609	No	0.9988
	Osmo: Low vs high	1	–5.037 to 0.4289	No	0.1028
	Size: Ctr vs small	2	–2.405 to 1.561	No	0.6416
	Temp: Ctr vs high	2	–2.067 to 1.073	No	0.4785
3*C*	Food: 0 vs 1	1	–168.3 to 323.3	No	0.6736
	Food: 0 vs 4	1	–276.1 to 190.3	No	0.8709
	Food: 1 vs 4	1	–353.6 to 112.8	No	0.3699
	Osmo: Ctr vs low	1	–448.7 to –9.334	Yes	0.0406
	Osmo: Ctr vs high	1	5.853 to 341.4	Yes	0.0422
	Osmo: Low vs high	1	192.3 to 612.9	Yes	0.0005
	Size: Ctr vs small	2	–96.12 to 287.7	No	0.288
	Temp: Ctr vs high	2	–173.1 to 196.4	No	0.8855
3*D*	Food: 0 vs 1	1	–890.9 to 180.7	No	0.2575
	Food: 0 vs 4	1	–594.9 to 429.8	No	0.9638
	Food: 1 vs 4	1	–198.1 to 743.2	No	0.3624
	Osmo: Ctr vs low	1	–398.8 to 148.8	No	0.4752
	Osmo: Ctr vs high	1	–285.7 to 221.3	No	0.9411
	Osmo: Low vs high	1	–160.7 to 346.3	No	0.6139
	Size: Ctr vs small	2	–189.6 to 358.8	No	0.497
	Temp: Ctr vs high	2	–430.9 to 270.0	No	0.5946
3*E*	Food: 0 vs 1	1	–20.65 to 14.51	No	0.8669
	Food: 0 vs 4	1	–31.19 to 3.966	No	0.1246
	Food: 1 vs 4	1	–29.33 to 8.249	No	0.2875
	Osmo: Ctr vs low	1	–18.11 to 16.66	No	0.9932
	Osmo: Ctr vs high	1	–9.921 to 18.47	No	0.7082
	Osmo: Low vs high	1	–12.39 to 22.38	No	0.7294
	Size: Ctr vs small	2	–4.836 to 10.90	No	0.406
	Temp: Ctr vs high	2	–4.654 to 17.22	No	0.2095
3*F*	Food: 0 vs 1	1	–878.5 to 168.3	No	0.1957
	Food: 0 vs 4	1	–583.0 to 417.9	No	0.8911
	Food: 1 vs 4	1	–187.2 to 732.3	No	0.2734
	Osmo: Ctr vs low	1	–23.83 to 22.78	No	0.998
	Osmo: Ctr vs high	1	–25.81 to 12.84	No	0.6477
	Osmo: Low vs high	1	–28.53 to 16.61	No	0.7608
	Size: Ctr vs small	2	–6.952 to 12.37	No	0.536
	Temp: Ctr vs high	2	–4.654 to 17.22	No	0.2095
3*G*	Food: 0 vs 1	1	–0.6621 to 3.852	No	0.1787
	Food: 0 vs 4	1	–1.813 to 2.470	No	0.908
	Food: 1 vs 4	1	–3.408 to 0.8747	No	0.2816
	Osmo: Ctr vs low	1	–6.687 to –1.087	Yes	0.0066
	Osmo: Ctr vs high	1	–0.5830 to 4.411	No	0.1499
	Osmo: Low vs high	1	3.165 to 8.437	Yes	<0.0001
	Size: Ctr vs small	2	–1.356 to 3.915	No	0.3006
	Temp: Ctr vs high	2	–0.8085 to 4.736	No	0.1378
3*H*	Food: 0 vs 1	1	–9.974 to 2.307	No	0.2484
	Food: 0 vs 4	1	–6.661 to 4.990	No	0.919
	Food: 1 vs 4	1	–2.828 to 8.823	No	0.3722
	Osmo: Ctr vs low	1	–389.9 to 139.9	No	0.4301
	Osmo: Ctr vs high	1	–382.1 to 229.7	No	0.7785
	Osmo: Low vs high	1	–257.1 to 354.7	No	0.901
	Size: Ctr vs small	2	–3.063 to 4.773	No	0.6283
	Temp: Ctr vs high	2	–6.933 to 3.432	No	0.4402
4*D*	High vs Ctr	2	5.575 to 43.27	Yes	0.0193

Method 1 indicates ordinary one-way ANOVA, Tukey’s multiple comparison test, and method 2 indicates unpaired *t* test. The four conditions used were food (Food), osmolarity (Osmo), size (Size), and temperature (Temp). Control medium (ctr).

### Code accessibility

All code is available as Extended Data 1. The MATLAB code was used to analyze neural and muscular activity in Figs. 2–4.

10.1523/ENEURO.0539-19.2020.ed1 Extended Data 1 Supplementary Code. Download Extended Data 1, ZIP file.

## Results

### *Hydra*’s contractile behavior affected by media osmolarity

*Hydra* has a small repertoire of highly stereotypical behaviors (Han et al., 2018). One of the most noticeable ones are spontaneous periodic contractions, known as “contraction bursts” (Wagner, 1905; Reis and Pierro, 1955; Passano and Mccullough, 1964). Possible roles of contractions by *Hydra* include foraging, protection by retraction (Miglietta et al., 2000; Swain et al., 2015), food digestion (Shimizu and Fujisawa, 2003), and excreting excess water from the body (Macklin et al., 1973). Another common behavior of *Hydra* is locomotion, i.e., translocation of the foot from one place to another. This is initiated by “foot detachment,” where the basal disk detaches from a substrate’s surface (Rodrigues et al., 2016).

**Figure 1.**
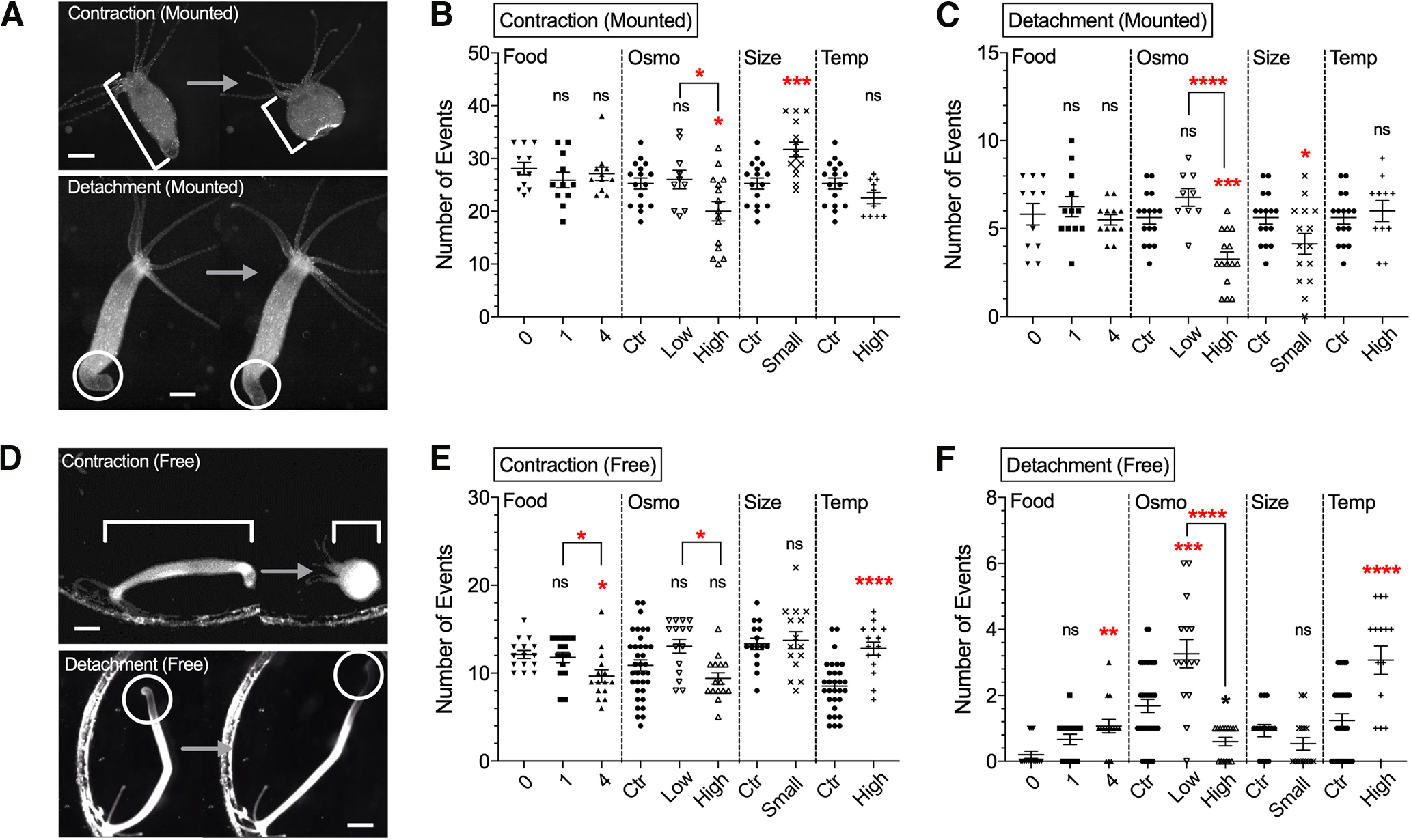
Effect of experimental conditions on contractions and locomotion behavior. Data from mounted preparations in ***A***–***C*** and from 1-h freely moving *Hydra* in ***D***–***F***. ***A***, Upper images, Changes in body length during longitudinal contractions. Lower images, Foot detachment. Scale bar, 500 μm. Number of contractions (***B***) and foot detachments (***C***) were counted. ***D***, Upper images depict changes in body length during longitudinal contractions. Lower images depict foot detachment followed by locomotion. Scale bar, 1 mm. Number of contractions (***E***) and foot detachment/locomotion (***F***) were counted. The four conditions used were food (Food), osmolarity (Osmo), size (Size), and temperature (Temp). Control medium (ctr). Error bars shown as mean ± SEM, with symbol marks denoting data points from individual *Hydra* (*N *=* *9–16 for ***B***, ***C***; *N *=* *15–30 for ***E***, ***F***). Tukey’s multiple comparisons tests were performed following one-way ANOVA for osmolarity experiment, and Student’s *t* test was performed for others: *n*s ≥ 0.05; *p <* *0.05, **p <* *0.01, ***p <* *0.001, ****p <* *0.0001.

We first tested how these two simple behaviors of *Hydra* were affected by various physiological and environmental conditions. Conditions chosen included amount of food, osmolarity or temperature of media, and the size of an animal. For the amount of food, *Hydra* was starved for 1 d before an experiment. For each condition, the frequency and duration of contractions and foot detachments were measured. In mounted preparations, where specimens are place in a microscope chamber with a spacer, osmolarity or body size robustly changed the frequency of contractions (Fig. 1*A–C*; see Materials and Methods). High-osmolarity media significantly decreased the frequency of contractions compared with control (Fig. 1*B*, *p* = 0.0380) or low-osmolarity conditions (Fig. 1*B*, *p* = 0.0367). Similarly, high-osmolarity media significantly decreased the number of foot detachments compared with control (Fig. 1*C*, *p* = 0.0003) or low-osmolarity conditions (Fig. 1*C*, *p* < 0.0001). Also, smaller size *Hydra* had more contractions (Fig. 1*B*, *p* = 0.0008) but fewer foot detachments (Fig. 1*C*, *p* = 0.0378).

As mounting restricts *Hydra* behavior, because of compression of body between glass coverslips, we also imaged freely moving *Hydra* under widefield illumination in the same conditions (Movie 1). Consistent with results in mounted preparations (Fig. 1*B*,*C*), in free moving animals, high osmolarity also decreased the number of contractions compared with low osmolarity (Fig. 1*E*, *p* = 0.0100) and the number of foot detachments, compared with control (Fig. 1*F*, *p* = 0.0134) or low-osmolarity conditions (Fig. 1*F*, *p* < 0.0001). But, unlike mounted preparations, well-fed (four shrimp per day) *Hydra* did not show any difference in behavior, comparing with control conditions. (Fig. 1*B*, *p* = 0.8506 for contractions; Fig. 1*C*, *p* = 0.8980 for detachments). Also, in well-fed freely moving *Hydra*, the number of contractions decreased (Fig. 1*E*, *p* = 0.0164), while the number of foot detachments increased (Fig. 1*F*, *p* = 0.0014). High temperature also increased contractions (Fig. 1*E*, *p* < 0.0001) and foot detachments (Fig. 1*F*, *p* < 0.0001) in freely moving animals. Overall, osmolarity was the only parameter that robustly changed behavior in both freely moving and mounted specimens. As motor behaviors must be generated as a result of contractile force derived from muscle, we next assessed how these changes in behaviors are accounted for the activity of muscle cells. For these experiments, we used exclusively mounted preparation, as it is yet not feasible to image and reconstruct the activity of neurons and muscle cells in freely moving animals.

Movie 1. Freely moving *Hydra* in control media. Animals were allowed to move freely in a Petri dish. Video was taken at 2 Hz and sped up 40-fold. Scale bar, 1 mm. 10.1523/ENEURO.0539-19.2020.video.1

### Bidirectional effects of osmolarity on ectodermal muscle activity

**Figure 2.**
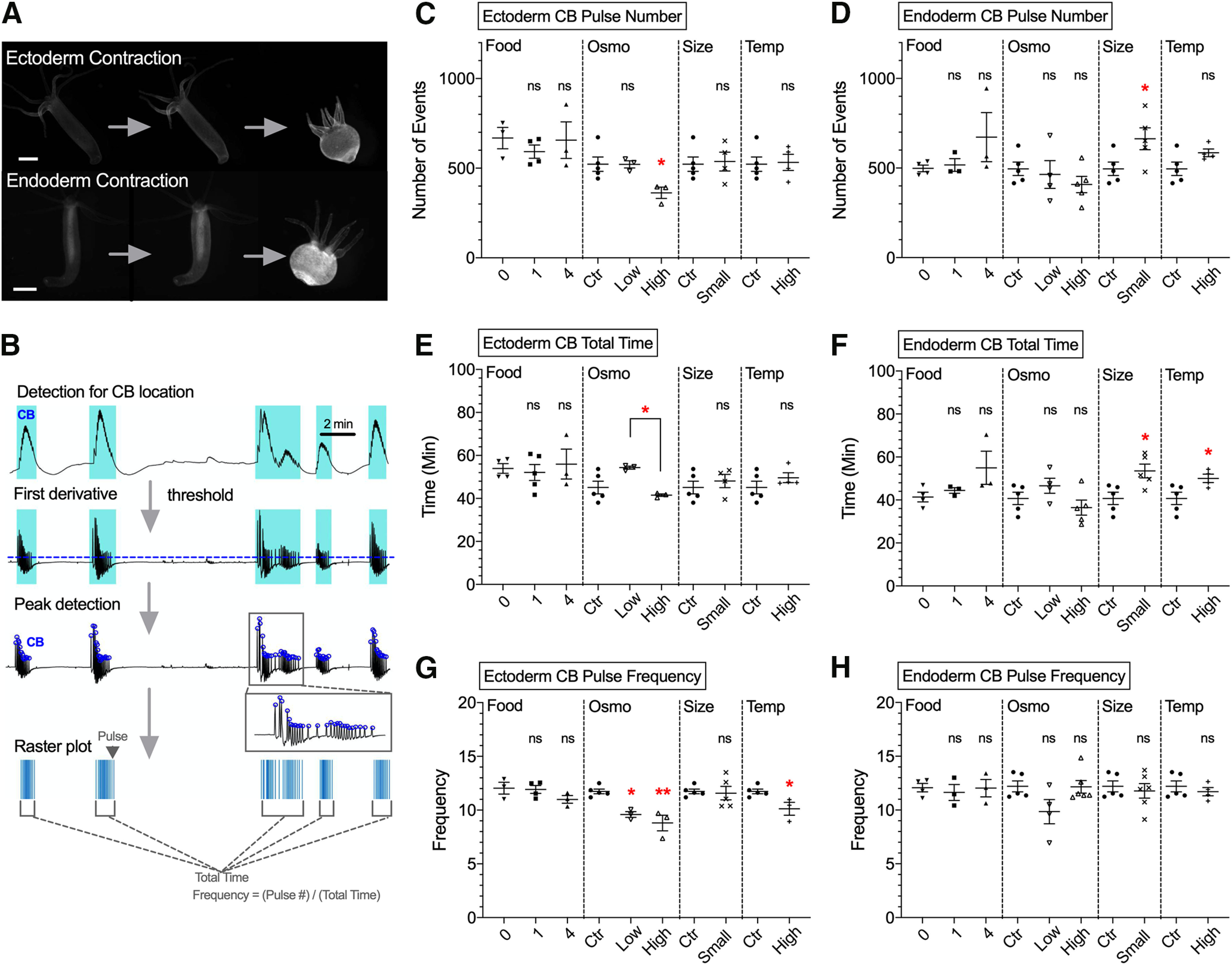
Effect of experimental conditions on ectoderm and endoderm muscle activity. ***A***, upper images, Measurements of contractions in *Hydra* expressing GCaMP6s in ectoderm muscle. Lower images, contractions in *Hydra* expressing GCaMP6s in endoderm muscle. Scale bar, 500 μm. ***B***, Schematic summarizing steps to detect peaks of CB pulses from raw traces extracted from 2-h calcium imaging movies. RP1 pulses were not present in muscle activity. ***C***–***H***, Each type of response was analyzed with four variables: (***C***) ectoderm CB pulse number; (***D***) endoderm CB pulse number; (***E***) ectoderm CB total time; (***F***) endoderm CB total time; (***G***) ectoderm CB total time; (***H***) endoderm CB total time. The four conditions used were food (Food), osmolarity (Osmo), size (Size), and temperature (Temp). Control medium (ctr). Error bars are shown as the mean ± SEM, with symbol marks denoting data points from individual *Hydra* (*N *=* *3–6). Tukey’s multiple comparisons tests were performed following one-way ANOVA for osmolarity experiment, and Student’s *t* test was performed for others: *n*s ≥ 0.05; *p <* *0.05.

*Hydra*’s body is composed of two layers of cells: ectodermal and endodermal epitheliomuscular tissues. Both epithelia are separated by an extracellular matrix called mesoglea. Inside these epithelial layers, there is a gastrovascular cavity that functions as a both gut and vasculature and carries nutrients to the entire body (Shimizu and Fujisawa, 2003). Both ectoderm and endoderm epitheliomuscular tissues generate action potentials (Dupre and Yuste, 2017; Szymanski and Yuste, 2019), which likely propagate through gap junctions (Westfall et al., 1980). These muscle cells contract in a calcium-dependent manner through myonemes, intracellular muscle processes that run longitudinally along the ectoderm and radially in the endoderm (Otto, 1977). Thus, *Hydra* generates motor behavior such as contractions and elongations by coordinating the activity of these two layers of muscle (Szymanski and Yuste, 2019). However, how their activity is affected by physiological and environmental conditions has not been characterized. To test the effect of environmental manipulations on muscle activity, we used transgenic *Hydra* that express genetically-encoded calcium indicator GCaMP6s in every ectoderm or endoderm muscle cell (Szymanski and Yuste, 2019). With these transgenic animals, 2-h-long calcium imaging sessions were conducted (Movie 2) to explore how each physiological or environmental condition changes muscle activity (Fig. 2*A*).

Movie 2. Ectoderm muscle activity in control media. The animal was allowed to move between coverslips in mounted configuration. Video was taken at 2 Hz and sped up 20-fold. Scale bar, 500 μm. 10.1523/ENEURO.0539-19.2020.video.2

Widespread activation of the entire body musculature was observed when *Hydra* contracted, as described previously (Szymanski and Yuste, 2019), with transient calcium increases that synchronously occurred in the entire muscle tissue. These activations usually appeared as a burst during each contraction event, faithfully reflecting behavioral CBs (Passano and McCullough, 1963, 1964). To analyze the spatiotemporal dynamics of these muscle pulses and bursts, we used a computer program to semi-automatically detect events from whole-body fluorescence intensity measurements (Fig. 2*B*). In agreement with behavioral data (Fig. 1), in ectoderm muscle tissue, high osmolarity decreased the number of pulses (Fig. 2*C*, *p* = 0.0356), burst duration (Fig. 2*E*, *p* = 0.0273), and frequency (Fig. 2*G*, *p* = 0.0017), as compared with low osmolarity. In contrast, we detected no change in endoderm muscle activity in response to osmolarity changes, although increases in endoderm muscle activity were observed during contractions, and changes of that baseline rate was also observed in smaller *Hydra*, or with increased temperature (Fig. 2*D*,*F*,*H*).

We concluded that osmolarity altered ectodermal muscle activity in the same way as it changed contractile behavior but did not affect endodermal muscle. This is consistent with the hypothesis that ectodermal muscle generates CBs in the animal, responding to medium osmolarity. To search for the origin of their response, we then examined the neural activity, presumable controlling of this muscle activation.

### Bidirectional effect of osmolarity on CB neuronal circuit activity

*Hydra*’s nerve nets lie at the base of both ectodermal and endodermal epithelial layers (Sarras et al., 1991) and are divided functionally into non overlapping circuits (Dupre and Yuste, 2017). Two of such circuits are the CB and RP1 networks (Dupre and Yuste, 2017). These circuits activate in synchronous and oscillatory manner during *Hydra*’s spontaneous contraction (CB) or during elongation (RP1; Passano and McCullough, 1963; Rushforth and Burke, 1971; Dupre and Yuste, 2017). However, while these circuits likely have a combination of sensory and motor neurons, the exact role of these cells is still unclear. Similar to bilaterian species, the cnidarian *Hydra* has neuromuscular junctions (Chapman et al., 2010), and there is evidence suggesting direct interaction of muscle cells and neurons. First, gap junctions are found between muscle cells and neurons (Westfall et al., 1980). Second, *Hydra* contractions are greatly reduced after chemically eliminating neurons (Campbell et al., 1976), suggesting that muscle activity in *Hydra* are initiated and coordinated by neurons. We therefore set out to study neural activity in *Hydra* to account for the observed changes in the muscle activity and behavior under different conditions.

**Figure 3.**
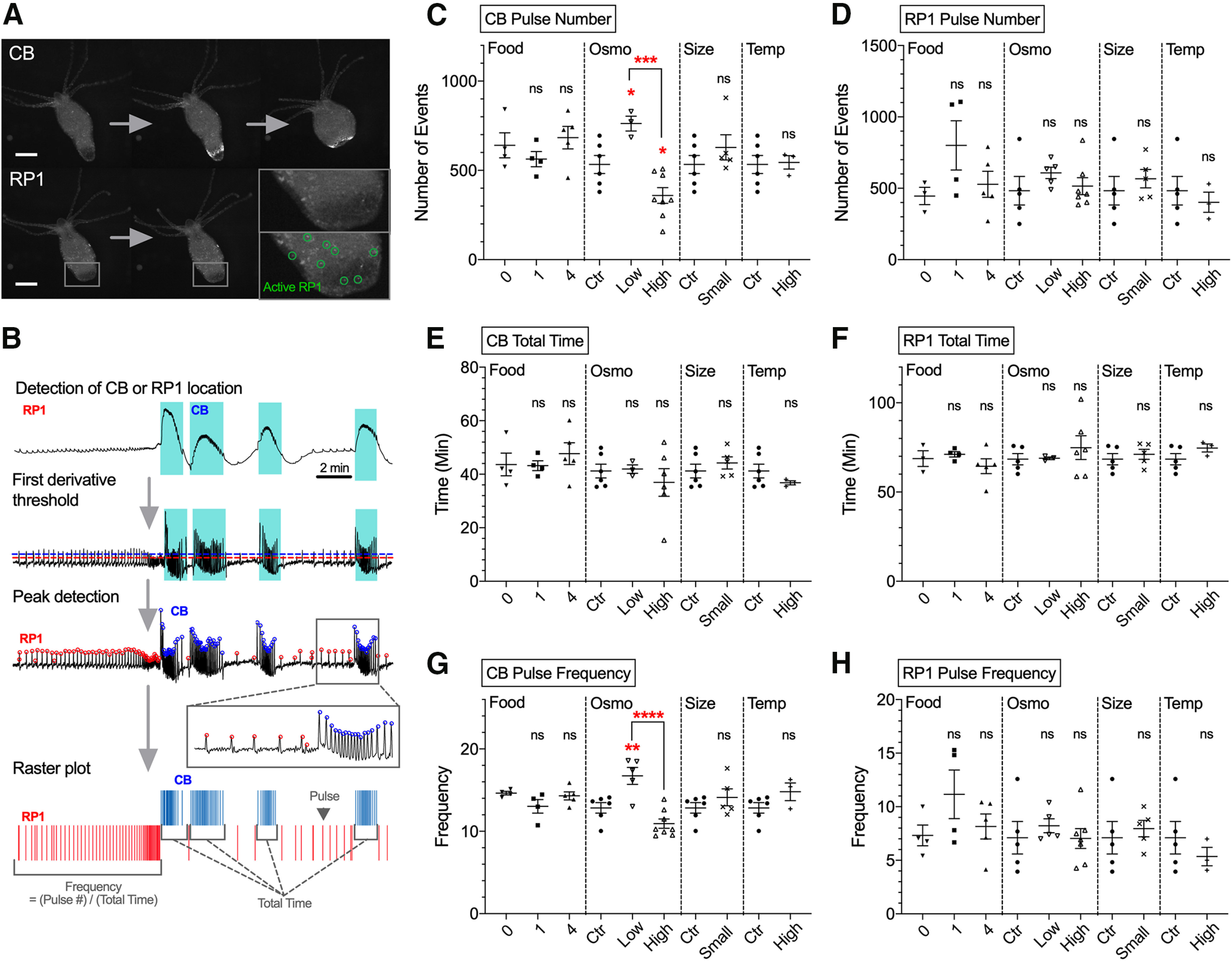
Effect of experimental conditions on neuronal activity. ***A***, upper images, Activation of CB neurons. Lower images, Activation of RP1 neurons. Scale bar, 500 μm. ***B***, Schematic summarizing steps to detect peaks of CB and RP1 pulses from raw traces extracted from 2-h calcium imaging. ***C***–***H***, Analysis of parameters: (***C***) CB pulse number; (***D***) RP1 pulse number; (***E***) CB total time; (***F***) RP1 total time; (***G***) CB pulse frequency; (***H***) RP1 pulse frequency. The four conditions used were food (Food), osmolarity (Osmo), size (Size), and temperature (Temp). Control medium (ctr). Error bars are shown as the mean ± SEM, with symbol marks denoting data points from individual *Hydra* (*N *=* *3–8). Tukey’s multiple comparisons tests were performed following one-way ANOVA for osmolarity experiment, and Student’s *t* test was performed for others: *n*s ≥ 0.05; *p <* *0.05, **p <* *0.01, ***p <* *0.001, ****p <* *0.0001.

Similarly to muscle imaging experiments (Fig. 2), 2-h calcium imaging sessions were conducted in mounted preparations using *Hydra* expressing GCaMP6s in the entire nerve net (Movie 3; Fig. 3*A*; Dupre and Yuste, 2017). Then, the spatiotemporal dynamics of the CB and RP1 pulses for the entire neuronal populations were semi-automatically extracted using a computer program from whole-body fluorescence measurements (Fig. 3*B*), and events frequencies were calculated. Results showed that low osmolarity increased the number of neuronal CB pulses compared with control, while high osmolarity decreased them (*p* = 0.0422) compared with control or low osmolarity (*p* = 0.0005; Fig. 3*C*), with no significant change in neuronal CB burst duration (Fig. 3*E*). In addition, high osmolarity decreased CB pulse frequency, compared with low osmolarity (*p* < 0.0001), while low osmolarity increased CB pulse frequency compared with controls (*p* = 0.0066; Fig. 3*G*). Oher experimental conditions (food, temperature, and body size) did not significantly alter the activity of CB neurons. These results indicate that CB neural activity is inversely proportional to osmolarity: lower osmolarity increases neuronal CB activity while higher osmolarity decreases it.

Movie 3. Neural activity in control media. The animal was allowed to move between coverslips in mounted configuration. Video was taken at 2 Hz and sped up 20-fold. Scale bar, 500 μm. 10.1523/ENEURO.0539-19.2020.video.3

In contrast to these results in CB neurons, none of the condition altered the activity of RP1 neurons, thought to be responsible for body elongation (Fig. 3*D*,*F*,*H*; Dupre and Yuste, 2017). These results suggest that the activity of RP1 neurons are not affected by the environmental conditions tested. Overall, osmolarity consistently altered contractions, ectoderm muscle activity, and CB neuronal activity, with hypo-osmolarity leading to increases and hyperosmolarity to decreases in all these three physiological outputs. These results suggest that the neuronal CB circuit is the origin on the osmolarity response and the generation of CB muscle activity and CB contractions.

## Discussion

In this study, we examined the effect of internal and external experimental factors on the contractile behavior and activity of muscle and neural tissue of *H. vulgaris*. We established imaging and analysis methods to measure the activity of all neuron and muscle cells during behavior in mounted preparations, under different physiological and environmental conditions. Among the conditions tested (amount of food, osmolarity or temperature of media, and size of animal), osmolarity consistently affected three functional readouts, in both free behaving and mounted preparations: contractile behavior, ectoderm muscle activity, and neural activity of the CB circuit. For foot detachments, ectodermal muscle CB duration and neuronal CB frequency, these effects were bidirectional, inversely related to osmolarity. Thus, *Hydra* appears to respond to osmolarity by specifically changing its neural and muscular activity, which presumably then changes behavior.

In both mounted and freely moving preparations, the number of contractions of *Hydra* in high osmolarity significantly decreased compared with low osmolarity (Fig. 1*B*,*E*), consistent with previous behavioral findings (Benos and Prusch, 1973). Changes of *Hydra* behavior with osmolarity are thought to be triggered by increased water accumulation in *Hydra*’s gastrovascular cavity, causing *Hydra* to swell. As *Hydra* cells are highly permeable to water (Lilly, 1955), water could follow the concentration gradient between media (∼5 mOsm/l) and *Hydra* tissue (∼120 mOsm/l), accumulating in the gastrovascular cavity (∼60 mOsm/l), which serves as an excretory pathway in these basal metazoans that lack excretory systems (Benos and Prusch, 1972). Furthermore, previous reports have suggested that the speed of water accumulation in *Hydra* tissues depends on osmolarity (Kücken et al., 2008; Soriano et al., 2009). Using regenerating hollow spheres of *Hydra* tissue fragments, made of two epithelial layers as in intact *Hydra*, the speed of sphere swelling because of water accumulation decreased linearly with increasing osmolarity (Kücken et al., 2008; Soriano et al., 2009). Our results are in excellent agreement with this previous work, demonstrating concomitant changes in the ectodermal muscle and CB neuronal circuits, thus providing a neurobiological pathway that mediates this osmolarity reflex. By contracting its body, Hydra would be “wringing” itself periodically, eliminating excess water from its cells.

What are the mechanisms by which *Hydra* alters the contractions with osmolarity? One possibility is a mechanosensory system that could sense tissue pressure. Mechanosensory responses in *Hydra* have been characterized in cnidocytes (Kass-Simon and Scappaticci, 2002), which use neurons to regulate their activation. *Hydra* is expected to express a set of potential osmoregulatory genes and mechanosenseory receptor genes such as TRP channels, integrin (Pedersen et al., 2011; Siebert et al., 2019), and it will be interesting to examine the functions of these proteins in regulating neuronal and muscular activity during behavior.

**Figure 4.**
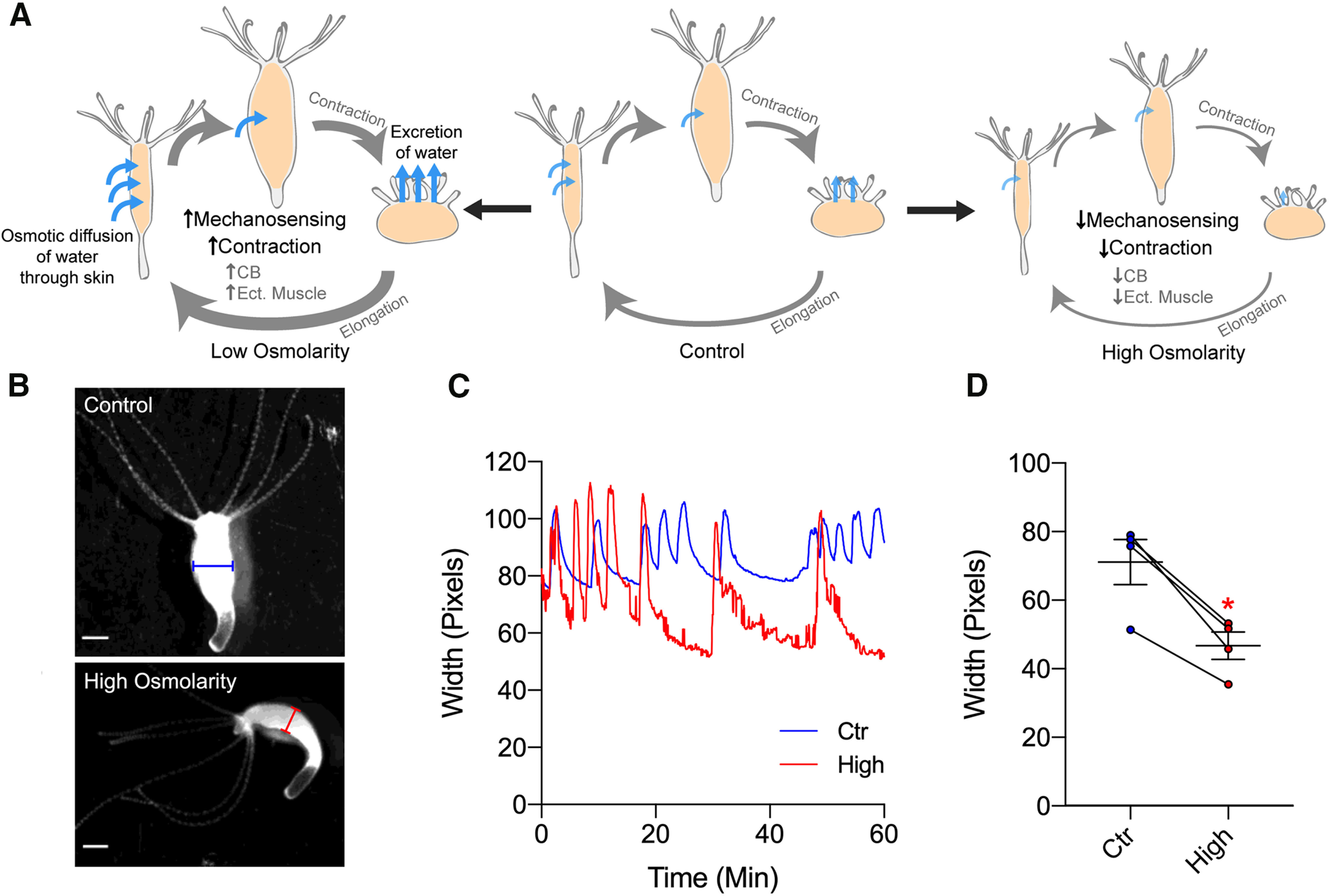
Proposed model and effect of osmolarity on body width. ***A***, Schematic model depicting how *Hydra* changes body width depending on osmolarity. Light-blue arrows indicate the direction and speed of water accumulation, which swells *Hydra*’s body and activate mechanosensory system and contractions. ***B***, Representative images showing width of *Hydra*’s body column at the end of elongation cycle, under control media (blue, above) or high-osmolarity solution (red, below). ***C***, Representative traces showing changes in width over time under control media (blue) or high-osmolarity solution (red). ***D***, Width of body column in control media (blue, 70.962 ± 6.560) or high-osmolarity solution (red, 46.540 ± 4.036). Line depicts the same animal in each condition. Error bars are shown as the mean ± SEM, with symbol marks denoting data points from individual *Hydra* (*N *=* *4). Student’s *t* test was performed: *p <* *0.05.

We propose the following model (Fig. 4*A*): *Hydra* undergoes a spontaneous cycle of elongation and contraction. In low osmolarity, this cycle speeds up because of increases in water accumulation and activation of mechanosensory receptors in the tissue. In contrast, in high osmolarity, this cycle slows down because of decrease in water accumulation and lesser activation of mechanosensory receptors. As a first test of this model, we found that high-osmolarity solution (50 mm sucrose) significantly shortens the width of the body column, as if water accumulation was indeed reduced (Fig. 4*B-D*). According to our results, body contractions would be generated by ectodermal muscles, themselves under the control of CB neurons. But while responses were indeed altered in an osmolarity-dependent manner in both CB neurons and ectoderm muscle tissue, our data also showed no change in endoderm muscle activity with osmolarity. CB neurons localize within the ectoderm layer, so their activity and those of ectoderm muscle are mutually consistent (Figs. 2, 3). Thus, CB neurons could be the motor neurons that forms synapse onto ectodermal muscle cells and activate them. On the other hand, endoderm muscle appears not to contact CB neurons or ectoderm muscle (Rushforth and Burke, 1971; Dupre and Yuste, 2017), behaving as a separate system, somehow unaffected by changes in osmolarity. Future experiments could examine ectoderm and endoderm muscle activity together, with simultaneous calcium imaging of both tissues with two different color indicators. Also, simultaneous imaging of neurons and muscle cells using transgenic *Hydra* that expresses different color calcium sensors in both sets of cells could explore the relationship between CB neurons and ectoderm muscle. Furthermore, future analysis based on the activity of individual neurons, which still requires the development of robust tracking software, could reveal additional neuronal mechanisms of how osmolarity altered various behavior at single-neuron resolution.

We also found conditions that changed contractions in free behavior without altering neuronal or muscle activity in mounted preparations. Although they were not the direct object of our study, as they did not occur in conditions where we could perform calcium imaging of the neuronal and muscle cells, it is still interesting to comment on them. For instance, during free behavior, high temperature (30°C) increased the number of contractions and foot detachments (Fig. 1*E*,*F*). Above 25°C, *Hydra* activates heat shock protein pathways leading to apoptosis; 30°C is eventually lethal to *Hydra* (Bosch et al., 1988), so increased locomotion could reflect an escape behavior, likely absent in mounted preparations. We also found that well-fed freely behaving animal (four shrimp per day) had fewer contractions overall but increased locomotion, as measured by foot detachments (Fig. 1*E*). It is not clear what could be the physiological function of these behaviors and why these conditions did not alter the activity of neurons or muscles in mounted preparations. The activity of CB neurons and contractions is inhibited during *Hydra*’s feeding behavior, while the activities of CB neurons and contractions increased right after the feeding behavior (Grosvenor et al., 1996). In the current study, rather than measuring at the immediate effect by feeding, we tallied changes in behavior of *Hydra* that had been fed various amount of food constantly for a week, and the experiments were conducted after starving for 1 d. Therefore, our conditions were not exactly comparable to those of Grosvenor et al. (1996), and measurements revealed *Hydra* did not alter muscle or neuronal activity depending on their energy state. Finally, it also remains possible that the differences between free-behaving and mounted animals could be that mechanical restrictions of *Hydra* may have disrupted physiological responses of neurons and muscles to heat and food. This effect should be reexamined by imaging neurons and muscle activity of freely moving *Hydra*, perhaps with wide-field 3D high-speed scanning systems (Cong et al., 2017; Kim et al., 2017).

In summary, using *Hydra*, we measured and analyzed the activity of the entire neuronal and muscle tissue in an animal during behavior. We find that osmolarity controls the activity of a selective group of neurons and muscle cells, without affecting others, leading to changes in contractile behavior. This approach, measuring the entire neuronal and muscle activity during a simple behavior in an accessible preparation, could be used systematically in *Hydra* and other animals to understand how neuronal and muscle function generates behavior.

## Synthesis

Reviewing Editor: Upinder S. Bhalla, National Centre for Biological Sciences, Tata Institute of Fundamental Research; Decisions are customarily a result of the Reviewing Editor and the peer reviewers coming together and discussing their recommendations until a consensus is reached. When revisions are invited, a fact-based synthesis statement explaining their decision and outlining what is needed to prepare a revision will be listed below. The following reviewer(s) agreed to reveal their identity: Gabriele Kass-Simon, Marius Pachitariu.

Synthesis:

The authors examine neural and muscle activity of hydra under various conditions, using imaging to monitor selected subsets of neurons/muscle cells iin parallel. The reviewers agreed that this is an interesting model system, however they felt that the MS fell short in some respects.

1. The authors must motivate the paper with a better account of what is known about osmolarity-driven behavior in hydra. They must clearly state what are the new and unexpected findings of the current study.

2. There are several apparent contradictions from the current study with previous work, including from their own lab. These include the account of how contractile behaviours engage the CB neurons. The authors must discuss these and provide a rationale to reconcile them.

3. The current analysis does not appear to draw effectively on the very rich dataset from this study. The authors should look at more fine-grained relations between the neurons and the behaviors they elicit.

In addition, the reviewers had several detailed comments, which the authors should examine closely.

===============================================================================

Selected comments, reviewer 1:

The paper provides strong evidence that at least some of the osmoreceptors controlling hydra's responses to changes in environmental osmolarity are ectodermal rather than endodermal.

There were numerous typographical errors:

Abstract

1. Hypo-osmolar, not hipo-osmolar

2. Contraction burst neurons, not contraction bursts neurons

3. Penultimate paragraph: It should be made clear that only the ectodermal rhythmic potential system is meant and that the endodermal RP system is not included.

Introduction

pg. 1, Line 5 “focused” not “focus"

Pg. 2, last sentence: According to recent work of Dupre and Yuste (2017) there are three, not two, independent nerve nets in hydra.

:

Materials

Pg. 3 “brine shrimp “were” not “was"

Results

Pg. 6, penultimate paragraph,

1. first sentence: “where” not “were”.

2. fourth sentence:Remove the second “.” (Fig. 1B)

3. Sixth and seventh sentences: “contractions” not “contraction” and “detachments” not “detachment”.

*Pg. 7. Not clear whether feeding affected foot detachments in any way in mounted animals compared to controls in mounted (no statistics given).

*Pg. 7. Not clear whether the sentence concerning effect of high temperature refers on contractions and foot detachments refers to mounted or freely moving animals.

(see also last comment in the discussion)

Pg. 9 “hypo-osmolarity” not hipo-osmolarity

Discussion

Pg. 11. five lines from bottom of the paragraph: Statement that endoderm muscles do not contact CB neurons or muscles is not completely true. Ectoderm and Endoderm epitheliomuscle cells connect with each other via gap juctions, at least in the tentacle. (Hufnagel and Kass-Simon, 1976).

Pg. 11. Five lines from bottom , “on the other hand", not “hands"

Pg. 12 “head” not “heat"

Pg. 12. With regard to the effect of the degree of satiety (feeding condition) on CB behavior, it is worth comparing the findings of Grosvenor, et al. Chem. Senses, (1996) in which CBs were initially inhibited during actual feeding and then increased during post-feeding. The receptors for this behavior were determined to be ectodermal. It is not clear in the present paper, in which part of the feeding cycle, the data were taken. This should be noted also in the results and in the materials and methods.

-----------------------------------------------------

Selected comments, Reviewer 2

The study images neural and muscle activity of hydra in different conditions. Some of these conditions lead to behavioral changes, which are reflected in the neural activity and the muscles.

It was unclear to me from the introduction what was known and what wasn’t about hydra. In particular, the robust changes in behavior due to osmolarity are presented as novel findings, but the discussion mentions that this was shown before (Benos and Prusch, 1973). This seems to also apply to the other behaviors studied. What is novel perhaps is the observation that some behaviors in freely-swimming hydra cannot be replicated under fixation.

Given that changes in osmolarity drive behavior, it is not surprising that the muscles are involved, and that the neurons driving those muscles are involved. Is it surprising that the endoderm muscles are not involved? If so, please say so in the introduction and set some expectations for the reader. Are all the motor neurons expected to be CB and RP1? I think the introduction should set expectations that we do or don’t really know which neurons are motor.

Finally, the model the authors propose doesn’t seem particularly well related to this data. The authors did not measure water accumulation and the activation of mechanosensory receptors in order to say anything about how the hydra senses the osmolarity change. The main result here seems to be a correlative identification of which muscle types, and which neuron types drive contractions, but that can also be studied in spontaneous behaviors, which was already done in Dupre and Yuste, 2017 and Szymanski and Yuste, 2019. I am little surprised at the inconsistency with these previous studies, where contractile behaviors did not engage the CB neurons (the 2017 paper), and where contractile behaviors engaged both ectoderm and endoderm muscles (the 2019 paper).

I think the present paper would be significantly enriched by more analyses of the data. One could perhaps look at more fine-grained relations between the neurons and the behaviors they elicit, for example by comparing leftward vs rightward contractions, or any other kind of behavioral differences between contractions to see if they are correlated to the neural activity. Rather than averaging all CB and all RP1 neurons, one could perhaps do a single-unit analysis to see which neurons are engaged in which behaviors.

===================================================

## Author Response

Comments by Upinder S. Bhalla Reviewing Editor (summary of reviewers)

1. The authors must motivate the paper with a better account of what is known about osmolarity-driven behavior in hydra. They must clearly state what are the new and unexpected findings of the current study.

We have rewritten this section and hope to convey now the novelty and unexpected findings more accurately.

2. There are several apparent contradictions from the current study with previous work, including from their own lab. These include the account of how contractile behaviours engage the CB neurons. The authors must discuss these and provide a rationale to reconcile them.

We have address the two perceived contradictions now. One of them was a straight misreading of the literature by reviewer 2. The other one, the lack of endodermal muscle activation, is not contradictory with Szymanski et al., 2019. To be clear, we do see changes in endoderm muscle activity during contraction, but we don’t see a significant change in that baseline endodermal muscle activity with osmolarity. Osmolarity driven CB bursts were not studied in that previous paper.

3. The current analysis does not appear to draw effectively on the very rich dataset from this study. The authors should look at more fine-grained relations between the neurons and the behaviors they elicit.

We agree that single neuron analysis could significantly enhance this study, yet the ability to perform automatic tracking of the position of each neuron or muscle cell during the major behavioral contractions is still beyond our current abilities, because the movements are large comparted to the size of the animal, and are not isometric. Moreover, the calcium signal disappears when the neurons or muscle cells are not activity. We are working hard to develop these computational tools. Nevertheless, the “bulk” analysis that we perform is very sensitive, as it averages the responses of all neurons and muscle cells, and, as we show, can provided exquisitely selective information about their involvement in contraction bursts.

Comments from Reviewer 1

Abstract

1. Hypo-osmolar, not hipo-osmolar

2. Contraction burst neurons, not contraction bursts neurons

Corrected

3. Penultimate paragraph: It should be made clear that only the ectodermal rhythmic potential system is meant and that the endodermal RP system is not included.

Rewritten

Introduction

pg. 1, Line 5 “focused” not “focus” Corrected

Pg. 2, last sentence: According to recent work of Dupre and Yuste (2017) there are three, not

two, independent nerve nets in hydra.

There are 3 major network but actually at least 6 different networks.

Materials

Pg. 3 “brine shrimp “were” not “was” Corrected

Results

Pg. 6, penultimate paragraph,

1. first sentence: “where” not “were”. Corrected

2. fourth sentence:Remove the second “.” (Fig. 1B) Corrected

3. Sixth and seventh sentences: “contractions” not “contraction” and “detachments” not

"detachment”.

*Pg. 7. Not clear whether feeding affected foot detachments in any way in mounted animals compared to controls in mounted (no statistics given).

Corrected

*Pg. 7. Not clear whether the sentence concerning effect of high temperature refers on contractions and foot detachments refers to mounted or freely moving animals.

(see also last comment in the discussion) Rewritten

Pg. 9 “hypo-osmolarity” not hipo-osmolarity

Corrected

Discussion

Pg. 11 five lines from bottom of the paragraph: Statement that endoderm muscles do not contact CB neurons or muscles is not completely true. Ectoderm and Endoderm epitheliomuscle cells connect with each other via gap juctions, at least in the tentacle. (Hufnagel and Kass- Simon, 1976).

Corrected

Pg. 11. Five lines from bottom , “on the other hand", not “hands” Corrected

Pg. 12 “head” not “heat” We actually meant heat.

Pg. 12. With regard to the effect of the degree of satiety (feeding condition) on CB behavior, it is worth comparing the findings of Grosvenor, et al. Chem. Senses, (1996) in which CBs were initially inhibited during actual feeding and then increased during post-feeding. The receptors for this behavior were determined to be ectodermal. It is not clear in the present paper, in which part of the feeding cycle, the data were taken. This should be noted also in the results and in the materials and methods.

Thanks for the comments. As the reviewer pointed out, our text was ambiguous with respect to when the recording was done during actual feeding behavior. We compared groups fed various amount of food over a week, and experiment was done after one day of starvation. Therefore, to avoid ambiguity, we decided to use the term ‘food’ instead of ‘feeding’ throughout the manuscript. We have rewritten this section.

Comments from Reviewer 2

1) It was unclear to me from the introduction what was known and what wasn’t about hydra. In particular, the robust changes in behavior due to osmolarity are presented as novel findings, but the discussion mentions that this was shown before (Benos and Prusch, 1973). This seems to also apply to the other behaviors studied. What is novel perhaps is the observation that some behaviors in freely-swimming hydra cannot be replicated under fixation.

We have rewritten this section and clarify what was known from past publications. Previously, behavioral and electrophysiological observations showed that high osmolarity decreased the number of contractions (Benos and Prusch. 1973), that acute high temperature damaged Hydra (Bosch et al. 1988), and CB neuronal activity decreased during feeding behavior and increased immediately after feeding (Grosvenor et al. 1996). However, there was no study assessing behavioral and electrophysiological effects by various environmental and physiological conditions. It was unclear how Hydra react with different stimuli. To address this, we have conducted assays to systematically study behavior, and muscle and neuronal activity in various conditions. Our data revealed that osmolarity can affect behavior, ectodermal muscle and a specific neural circuits.

2) Given that changes in osmolarity drive behavior, it is not surprising that the muscles are involved, and that the neurons driving those muscles are involved. Is it surprising that the endoderm muscles are not involved? If so, please say so in the introduction and set some expectations for the reader. Are all the motor neurons expected to be CB and RP1? I think the introduction should set expectations that we do or don’t really know which neurons are motor.

We have rewritten these sections. As to the lack of activation of endodermal muscle, yes, we think that this is surprising. The number of contractions should decrease due to high osmolarity solution. Accordingly, ectodermal muscle that contract body-column and CB neurons that are active during contraction should decrease under high osmolarity solution. Szymanski et al., has reported that ecotodermal muscle and endodermal muscles coactivate during contraction (Szymanski et al., 2019). All of this is consistent with our findings. But, against our expectation, endodermal muscle did not change their activity. It is hard to draw a conclusion but we speculate now in the discussion that osmolarity may only ectoderm. Additional experiments such as the one using two color imaging of both ectoderm and endoderm will validate this data and gives more insights.

According to previous research, sensory neurons form synapses between epithelial cells and are thus expected to have a certain function as a motor neuron (Westfall 1973). Therefore, neurons could be multifunctional, and have a certain motor function. That being said, CB neurons seem to have the most robust motor function. A population of CB neurons is active during a body- column contraction, and the surrounding ectoderm muscle will contract when single CB neuron is active. When a population of RP1 neurons is active, we do not usually see muscle contraction. RP1 neurons were active during elongation (Dupre and Yuste 2017), and it is hard to tell that is due to RP1.

3) Finally, the model the authors propose doesn’t seem particularly well related to this data. The authors did not measure water accumulation and the activation of mechanosensory receptors in order to say anything about how the hydra senses the osmolarity change.

The change in the thickness of body (Fig. 4C and F) is consistent to the model, but it doesn’t demonstrate it. We think that adding the model to the paper serves to both summarize the work and provide a handle for future research. Future studies, perhaps using knockouts of osmoregulatory genes or mechanosensory genes (Siebert et al 2019), will add more information to this model.

The main result here seems to be a correlative identification of which muscle types, and which neuron types drive contractions, but that can also be studied in spontaneous behaviors,

which was already done in Dupre and Yuste, 2017 and Szymanski and Yuste, 2019.

Although our results are consistent with those two past studies, our focus was actually how osmolarity regulates muscle and neural activity. This was never done before. Our data indicates susceptibility to osmolarity and the ability to adapt to most conditions and provide a model that conceptually explains all the results.

I am little surprised at the inconsistency with these previous studies, where contractile behaviors did not engage the CB neurons (the 2017 paper),

The contractile behavior did engage CB neurons in the 2017 paper. and where contractile behaviors engaged both ectoderm and endoderm muscles (the 2019 paper).

This is also not contradictory, perhaps due to our unclear writing. To be clear, we do see changes in endoderm muscle activity during contractions (Figure 2F), but we don’t see a significant change in that baseline endodermal muscle activity with osmolarity. Osmolarity driven CB bursts were not studied in that previous paper.

"Do you need the model (authors did not measure water accumulation and the activation of mechanosensory receptors in order to say anything about how the hydra senses the osmolarity change)?"

As argued above, we think that the model adds to the paper. It provides both a summary and an initial test of the hypothesis. The model could help the reader follow the sometimes complex logic of the changes expected by hypo- or hyper osmolarity.

4) I think the present paper would be significantly enriched by more analyses of the data. One could perhaps look at more fine-grained relations between the neurons and the behaviors they elicit, for example by comparing leftward vs rightward contractions, or any other kind of behavioral differences between contractions to see if they are correlated to the neural activity. Rather than averaging all CB and all RP1 neurons, one could perhaps do a single-unit analysis to see which neurons are engaged in which behaviors.

As argued above, single neuron analysis is harder than it seems since Hydra moves significantly and isometrically during contractions. Moreover, the calcium signal disappears when the neuron or the muscle cell stops firing. We are developing better software for the automatic analysis of these type of date with single neuron resolution.
